# Opinions on hard-to-discuss topics change more via cohort replacement

**DOI:** 10.1017/ehs.2024.13

**Published:** 2024-04-08

**Authors:** Nicolas Restrepo Ochoa, Stephen Vaisey

**Affiliations:** 1 Department of Anthropology, University of California at Davis, Davis, CA, USA; 2 Department of Sociology, Duke University, Durham, NC, USA

**Keywords:** Cultural change, Cohort replacement, Cross-cultural comparisons

## Abstract

Cohort replacement – the replacement in a population of older cohorts by their successors who developed under different conditions – is an important process behind cultural change. Research on public opinion indicates that a large proportion of aggregate change is the result of cohort replacement rather than of individuals changing their minds. However, some publicly salient issues, like gay rights, appear to be exceptions. Why different issues show different patterns of change is not well understood. In this paper, we investigate whether opinions on sensitive – that is, hard to discuss – issues might change differently than opinions on less sensitive issues. We use data from the 1981–2020 World Values Surveys and newly collected data on the sensitivity of survey items to compare aggregate changes in public opinion on 56 survey items in eight countries. Our key finding is that survey items on more sensitive issues seem to change more through cohort replacement.

## Graphical abstract

**Figure:**
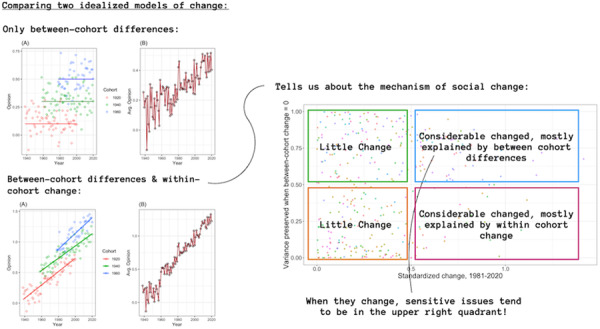

**Social media summary:** Cohort replacement explains more change in sensitive issues. Issues that are hard to talk about change more privately.

## Introduction

Much of the scholarly interest in culture lies in trying to understand how it changes. Cultural trends like secularisation (Berger, 2002; Chaves, 1994; Tormos, 2021) or the rise of (and backlash against) gender equality (Velasco, 2023) continue to receive sustained academic attention. Recent work on opinion change in the US suggests that most changes happen primarily – although not exclusively – through cohort replacement (Vaisey & Lizardo, 2016; Kiley & Vaisey, 2020). That is, culture usually changes as young people, who grew up in different social conditions, replace those that came before them. Nonetheless, beliefs about some salient issues, like gay rights, seem to be exceptions, where individuals appear to be changing their minds well into adulthood (Kiley & Vaisey, 2020; Tormos, 2021). This suggests that different beliefs might change via different underlying mechanisms. This is what we investigate in this paper.

We have two main goals. First, we examine whether there are systematic differences in patterns of change across variables, especially in relation to how sensitive – that is, how difficult to discuss – they are. Individuals do update their beliefs about particularly salient issues; however, these issues tend to be difficult to talk about because interlocutors are often firmly entrenched in their beliefs. The sensitivity of a topic might be a useful gateway to start asking how different beliefs change via different mechanisms. Second, following the work of Tormos and colleagues (Tormos, 2021; Tormos et al., 2023), we want to move beyond US data and examine cross-cultural variation in how different issues change. We approach this comparative work slightly differently, using methods that have – to date – mainly been implemented to explore change in the US context. In short, we want to dig deeper into the mechanisms that underpin change for different cultural issues, and we want to examine whether these mechanisms of change are consistent across contexts.

We investigate these questions using data from the World Value Survey (WVS) (Inglehart et al., 2000). Although the data are cross-sectional and therefore cannot directly answer questions about individual-level change, we can use them to adjudicate between different mechanisms that might account for population-level cultural change. To do this, we propose a straightforward method. We begin by positing an idealised model, where, after the critical period of youth, individuals do not change their beliefs. Under these assumptions, all cultural change can be explained by *between-cohort differences* and all we need to know to estimate a person's opinion on a given issue is to their year of birth. Then, we consider another model, where the average opinion of each cohort is allowed to change linearly over time. We contend that, when fitted to the WVS data, the relative explanatory power of these two models provides an indication of the mechanism that might be responsible for change for a given issue. If the proportion of the variance explained is relatively unchanged when we move from the first model to the second one, then this suggests that a given issue is changing primarily through cohort replacement. If the second model improves greatly on the first, then this would point towards the importance of *within-cohort change* in accounting for the trends.

To examine patterns of change across different types of cultural issues, we fit these models to 56 different variables, measured between 1981 and 2020, across eight countries. We select the countries – Argentina, Australia, Canada, Japan, Mexico, South Africa, Sweden, and the USA – based on completeness, seeking to cover the longest time-spans possible with this survey. We also choose to focus on variables that have been asked in all the waves of the WVS, and that cover a wide-range of topics and different levels of sensitivity, from the justifiability of euthanasia to whether imagination is a desirable attribute in children.

Our results lead to several key insights. We show that, consistent with previous work on opinion change over the past five decades, large differences are uncommon. Nonetheless, echoing work on attitudinal change in the USA (Kiley & Vaisey, 2020) and previous work using the WVS (Tormos, 2021), we find that the variable that has changed most consistently across the countries is related to attitudes about homosexuality. Furthermore, we show that cohort replacement explains a considerable portion of the variation in some of the variables that display the most linear change. Perhaps most relevant to our questions about the mechanisms of change, we see a pattern across the sensitivity of different cultural issues. We find that change in more sensitive topics can be explained mostly by *between-cohort differences*, and variation in less sensitive issues can be attributed more to *within-cohort change*. This provides some evidence for the claim that issues change through different mechanisms and provides a starting point for identifying which issues are more likely to change in different ways. All necessary code and data to reproduce this paper are available at: https://github.com/NicolasRestrep/sensitive_change.

## Cultural change and its elements

Cultural change has been a central preoccupation of social scientists. Recently, as new data sources with longer time series have become available, there has been a renewed interest in trying to understand cultural change quantitatively. This newer work has focused on linking individual mechanisms of belief updating to large-scale processes of change (Kiley & Vaisey, 2020; Keskintürk, 2021; Bartels & Jackman, 2014; Tormos, 2021).

When it comes to matters of beliefs and attitudes, *societies* do not change; *individuals* change, as does the composition of individuals in a population. The aggregation of those individual beliefs is what can we measure as shifts at the population level. Therefore theories of large-scale cultural change are – at their core – accounts of how individuals update their beliefs and habits.

Large-scale change can occur through several individual-level mechanisms. These mechanisms are generally classified into *age effects*, *period effects* and *cohort effects* (Fosse & Winship, 2019).

Age effects are reactions to one's personal ‘biography’. For instance, a citizen might veer away from direct action and radical politics as they accrue wealth and have more to lose in the case of a structural societal change (McAdam, 1989).

Period effects are the result of new information or events that affect an entire population at the same time. During a time for war, for example, we might expect individuals – across all age-groups – to change how they view the armed forces or their country in general.

Cohort effects are the enduring effects of certain historical moments – such at the Great Depression – that leave a mark on individuals who grow up under those conditions (Elder, 2018). These individuals, then, would have distinctive beliefs and attitudes that they would carry throughout their lives (Ryder, 1965; Elder, 2018; Fosse & Winship, 2023).

Disentangling these different sources of cultural change is a well-known challenge (Bell & Jones, 2013). In the framework of a standard regression with cross-sectional data, it is – strictly speaking – impossible. These three sources of variation are perfectly collinear because, if we know an individual's age and the current year, we also know precisely when they were born (Fosse & Winship, 2019). Although this is not the place to provide a full review of the work on age–period–cohort effects (cf. Fosse & Winship, 2019; Tormos, 2021), it suffices to mention that researchers have devised several strategies to disaggregate these three sources of change. Nonetheless, all strategies involve a kind of arbitrary compromise, like assuming quadratic age effects or binning cohorts into differently sized groupings (Vaisey & Lizardo, 2016).

Fortunately, given our questions, we do not need to disentangle all three types of effects. As we explain in the next section, the most important theoretical distinction is between a model that contains only between-cohort differences (i.e. cohort effects) and a model that includes within-cohort change (resulting from either age or period effects).

## Two models of individual-level change

There are two broad theoretical models of individual change. The first is the ‘settled dispositions’ model (Kiley & Vaisey, 2020; Underwood et al., 2022). This model posits that an individual's beliefs develop during a critical period of socialisation. After one's formative years, therefore, beliefs generally remain stable. This has the further implication that individuals raised in similar socio-historical contexts will share certain beliefs and attitudes that they carry throughout their lives (Elder, 2018; Gerber & Green, 1998; Ryder, 1965).

The second model is the ‘active updating’ model. It assumes that individuals remain open to revising their beliefs across the life course. This model is related to social theories that portray the self as continuously under construction (Gross, 2009). On this view, people are open to novel information – including biographical information gained through aging – and therefore sensitive to changes in their cultural and social contexts. This implies that cultural moments would play a much bigger role in shaping individuals’ attitudes (Tormos, 2021). In other words, individuals will reconsider their attitudes in light of the cultural trends and/or political movements happening at a particular historical moment. Visions of historical change as changes in the *zeitgeist* rely implicitly on the idea that individuals are attuned to the ‘spirit of the age’, ready to change their beliefs with the times.

Although no scholars believe that either model provides a complete account of change, attempts to compare the explanatory power of the two models has been at the centre of research about large-scale change for the past two decades (Vaisey & Lizardo, 2016; Tormos, 2021). In practical terms, Vaisey and Lizardo (2016) argue that the differences between these models can be boiled down to a rather simple question: *to predict a person's attitudes are we better off knowing the current year or their date of birth?*

Although, in practice, the question is not quite that simple, the settled dispositions and active updating models have different implications for the patterns we should expect to see at the population level over time. If the active updating model were the dominant process, we would expect cultural change to happen swiftly, following particular events or shocks (Tormos, 2021). For example, an unexpected economic downturn might lead the members of a group – regardless of age – to be more conservative in their financial choices. A series of catastrophic climate disasters might lead them to update their beliefs on human-induced climate change. In other words, exogenous changes will be reflected directly in aggregate cultural attitudes, as the population updates their attitudes in light of new information or new circumstances.

The settled dispositions model paints a rather different picture. It assumes that individuals beyond their formative years will be less swayed by exogenous changes. Thus, cohorts raised under unfavourable economic circumstances or during a climate crisis will develop attitudes based on these formative experiences even if the external environment later changes. Thus aggregate beliefs will only change as earlier cohorts, raised under different circumstances, die and are replaced (Ryder, 1965). Aggregate social change in this scenario will tend to be more gradual.

Recent work on belief change suggests that the settled dispositions model – although incomplete – is a better default model for explaining aggregate social change (Vaisey & Lizardo, 2016; Kiley & Vaisey, 2020; Underwood et al., 2022). Vaisey and Lizardo (2016), for instance, compare the explanatory power of both models in cross-sectional time-series data from the USA. They find that most beliefs remain relatively stable within cohorts, supporting the idea that aggregate change is best modelled as cohort succession. Analyses of panel data provides additional support that adults generally do not change their minds on issues over time (Kiley & Vaisey, 2020; Bartels & Jackman, 2014). As the settled dispositions model predicts, cohorts (and individuals) seem to remain generally stable on most issues over time.

## Variation in mechanisms of change across issues

The claim that cultural change occurs *primarily* through cohort replacement does not mean, of course, that this is the *only* mechanism of change. Recent work by Lersch (2023) shows that individuals do change in adulthood, even if observed changes are small in magnitude relative to persistent between-person differences. Kiley and Vaisey (2020) also show that there are certain issues where we observe durable change among adults. In the USA, for instance, there is evidence of intraindividual updating on beliefs about homosexuality, a particularly salient issue for the past few decades in the United States. Tormos (2021) also finds that, across the Organisation for Economic Co-operation and Development countries, cohorts also exhibit considerable change in their opinions towards homosexuality.

To this point, researchers have focused on general patterns, often counting the number of survey items for which different models provide better statistical fits to data (Vaisey & Lizardo, 2016; Kiley & Vaisey, 2020; Lersch, 2023). They also note exceptions (such as gay rights). However, to advance the science of cultural change, we have to investigate systematically *why* beliefs about different issues appear to change via different processes rather than telling just-so stories. What is it about some issues that makes beliefs about them more likely to change even in adulthood? And are there cross-cultural differences in what these issues are?

The number of attributes that might vary across topics is essentially infinite. However, we believe that the concept of *sensitivity* might allow us to get an initial handle on this problem. Campbell and Mace (this issue) define sensitive issues as issues that are difficult to talk about. In most surveys, questions vary a great deal in how sensitive they are, from the importance of friends in your life (perhaps not very sensitive) to the justifiability of suicide (perhaps quite sensitive). In the rest of the paper, we investigate whether answers to more sensitive questions show evidence for different change mechanisms than answers to less sensitive questions.

We believe that answers to questions about more sensitive issues will change more slowly (i.e. more by cohort replacement) than answers to questions about less sensitive issues. We believe this for two reasons. The first reason is psychological. The very sensitivity of the issues might mean that they constitute key elements in individuals’ worldviews. Beliefs on these issues might not open for discussion or revision.

The second reason is interactional. Sensitive issues are difficult to talk about and thus we talk about them less often or only with a few others. We gain less information about what other individuals believe, and thus external cues that might prompt reexamination are hard to come by. This would result in a scenario akin to pluralistic ignorance (Halbesleben & Buckley, 2004; O'Gorman, 1986), where individuals’ reticence to discuss certain topics precludes active conversations that might lead to attitudinal updating.

Both mechanisms lead to a similar conclusion: we should expect beliefs about more sensitive issues to change more slowly. This would mean that the changes we observe in beliefs on these topics at the aggregate level should be mostly attributed to cohort replacement.

## Methods

### Disentangling within-cohort and between-cohort differences

Our discussion above suggests that the goal is not disentangling the full range of age, period and cohort effects, but rather adjudicating the relative explanatory power of the two broad models of individual-level updating. This objective is simpler and more attainable. If the settled dispositions model is dominant, then we should expect most cultural change to be driven by differences between cohorts. In turn, if the active updating model is more explanatory, then we should see evidence of considerable changes within cohorts, as they age and as they experience new information and events. The central distinction, then, is between the relative importance of *between-cohort differences* and *within-cohort change*, with temporary *period effects* and biographical *age effects* subsumed in the latter.

**Figure 1.**
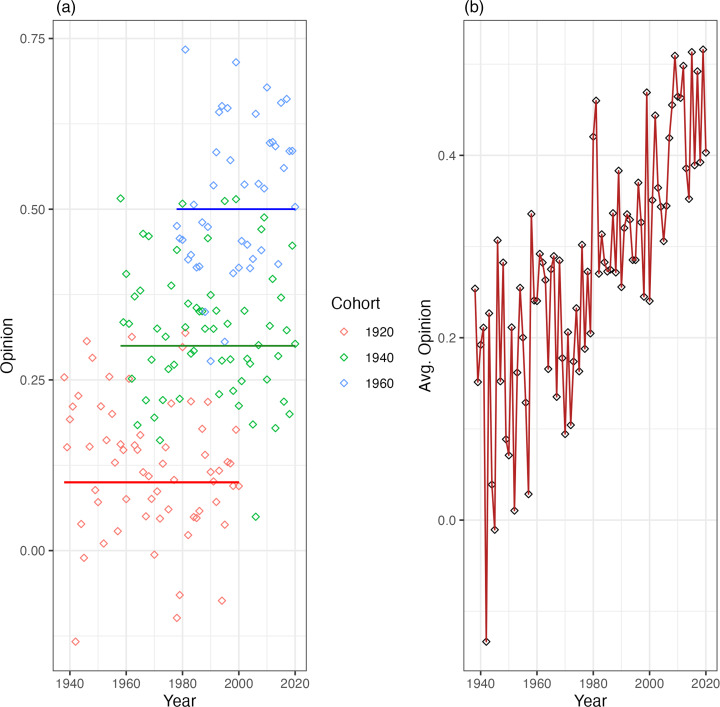
Cohort trends (a) and aggregate change (b) for an idealised model with no within-cohort changes.

To clarify the distinction between patterns of large-scale change mainly driven by *between-cohort differences* or *within-cohort change*, it is useful to envision two idealised models of aggregate change. First, imagine a scenario where, after the critical period of socialisation, cohorts have formed beliefs from which they do not deviate. If we were able to track the data by cohort it would look like overlapping horizontal lines, with different intercepts on the *y*-axis. Change, at the aggregate level, would look like a gradual shift towards the averages of the younger cohorts. Figure 1 illustrates both dynamics. In this case, knowing a person's year of birth would give us a good estimate of their opinion, in whatever year and at whatever age it what measured. Cohort differences would also explain all the variation in aggregate change, given that – in this idealised scenario – all change occurs through cohort replacement.

**Figure 2.**
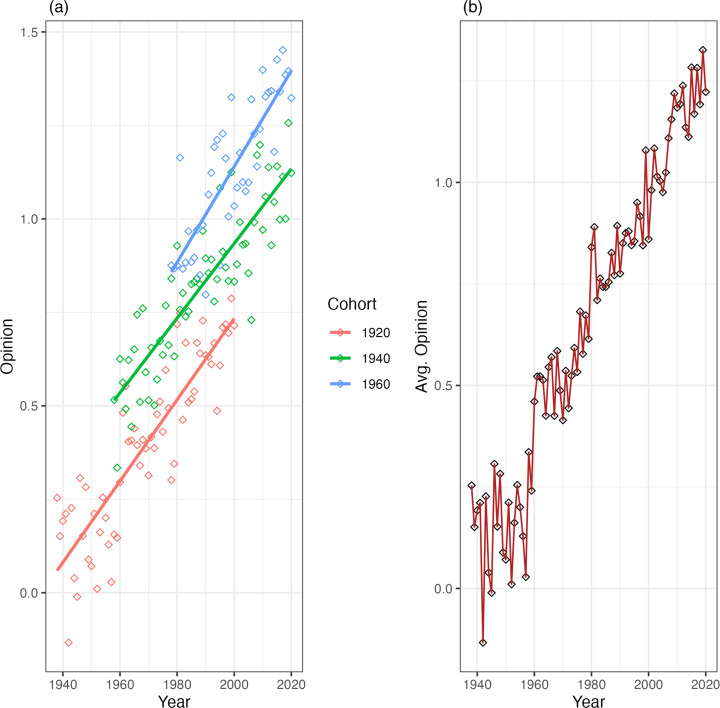
Cohort trends (a) and aggregate change (b) for an idealised model with both within-cohort changes & between-cohort differences.

Now, imagine another scenario where adults do update their beliefs, either because as individuals get older they tend to change their beliefs or because an issue has been particularly salient in public discussions. In other words, we would assume that there are, in addition to initial *between-cohort differences*, *within-cohort changes*, which can be either *period* or *age* effects (for our purposes, this distinction is unimportant). In this stylised example, we can imagine an issue – like attitudes towards homosexuality – that has become increasingly important in the public sphere since the middle of the twentieth century and where individuals seem to have updated their beliefs. Figure 2 shows this second example. Here we see *within cohort changes*, owing to common trends experienced by all members of the group. This, in turn, translates into much steeper cultural change at the aggregate level. Cultural change here is not only due to the overall differences between cohorts – and their replacement – but also due to changes in the same direction within cohorts.

**Figure 3.**
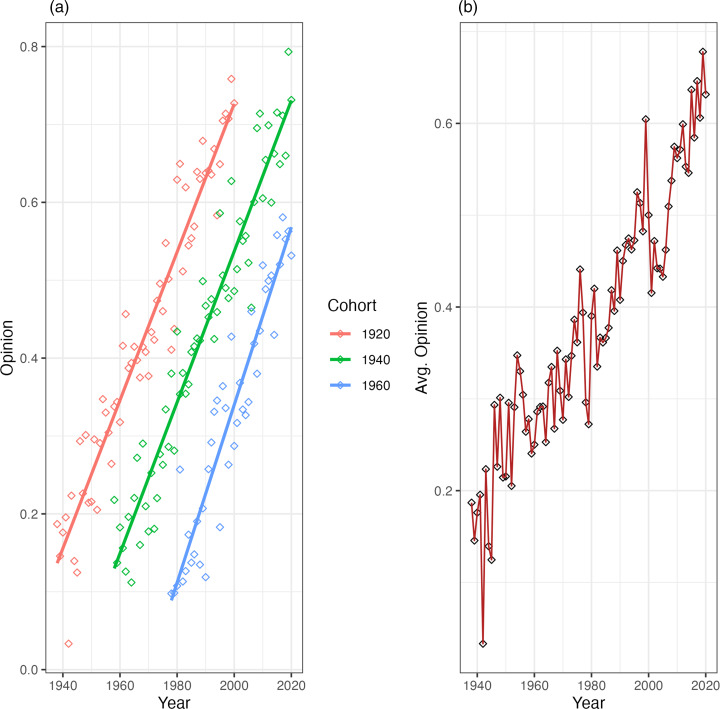
Cohort trends (a) and aggregate change (b) for an idealised model with no between-cohort differences.

A more extreme variant of this example would be one where all cohorts start from the same average opinion – regardless of the current year – and experience the same *within-cohort changes*. In other words, we can imagine a scenario where there are no initial *between-cohort differences* and all age-groups follow the same trends in opinion change. Figure 3 illustrates such a case.

Based on these idealised models, we propose a simple comparison that can help quantify the relative contribution of *within-cohort change* and *between-cohort differences*. We fit two models to the same data.

The first model is a regression where the outcome variable is regressed only on the cohort of each respondent:
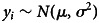

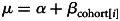


The second model adds a linear term for year (to allow for linear within-cohort change) and an interaction between cohorts and year, which allows every cohort to change in a different way:
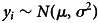

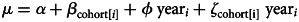


The use of linear within-cohort time trends here requires some justification. This assumption means that we are unable to capture within-cohort fluctuations that might be caused by temporary shocks – e.g. increased national pride during a national holiday or the Olympics – that leave no lasting effect on aggregate opinion. While we acknowledge that these fluctuations are a part of within-cohort *variation*, they cannot account for monotonic aggregate changes over time. When social scientists discuss cultural change, they typically mean *directional* change. In other words, we tend to be interested in variation that follows a trend, like secularisation or the liberalisation of attitudes about sexuality. Given that we are interested in how average opinions have changed in a single direction across our observation period, the linear assumption is theoretically justified. However, this does prevent us from saying anything about temporary changes, which can certainly be important for e.g. electoral outcomes in specific elections.

Comparing these two models can help us quantify the relative importance of between-cohort and within-cohort change. The second model – as a superset of the first – will always account for more of the variance in the outcome. Therefore dividing the variance explained by the first model by that accounted for by the second one, we have a measure that represents the proportion of variance explained that is preserved when only *between-cohort differences* matter – i.e. when we do not allow within-cohort change. We call this proportion *τ*:
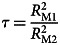


This measure may seem simple – perhaps too simple – but it captures the intuition behind comparing the models. Values of *τ* closer to 1 indicate that within-cohort changes add nothing to a model that includes only between cohort differences. For example, in the rather simple scenarios we discussed above, *τ* for the first case would be 0.98 and in the second case would be 0.72. For the third – admittedly extreme – case, *τ* would be 0.07. Almost all variance explained is preserved in the first case when we take the effect of survey year out of the model. In the second case, we lose information, as expected, because the model does not allow within-cohort changes. In the third case, almost *none* of the variance explained is preserved when we do not consider *within-cohort changes*, as these explain almost all the change in aggregate opinion. This simple metric, then, is a useful way to differentiate the mechanisms that underpin the large-scale opinion change in repeated cross-sectional data.

### Data

To compare mechanisms of change across different contexts and issues, we use the WVS (Inglehart et al., 2000). The WVS, which began in 1981, is a large-scale effort to collect comparable data on beliefs and attitudes across multiple countries. For each country and each wave, the WVS collects high-quality, nationally representative samples, and covers a wide range of questions from views on gender equality to socioeconomic indices. The survey, however, is not longitudinal, which means that we are unable to track any within-individual changes across time. However, it does allow us to examine trends in aggregate opinion across time for different countries.

Previous work has used the WVS to examine different mechanisms of social change to great effect (Tormos, 2021; Tormos et al., 2023). Our work builds on this literature in two ways. First, we build on conceptual debates that have focused primarily on the US context and apply them cross-culturally. Second, we compare trends not only across countries, but also across different types of variables to examine whether mechanisms of social change vary along these two axes.

Our method requires aggregate information in each country across a considerable time span. This is a challenge because not all countries feature in every wave of the WVS, and not all questions were asked in the times when we do have samples. For our analysis, we selected countries based on completeness – those for which we have the most measures over the longest period. This led us to select eight countries: Argentina, Australia, Canada, Japan, Mexico, South Africa, Sweden and the USA.

This is not a comprehensive or particularly diverse sample of countries. We are missing some of the world's most populous countries – India and China – and we do not have a majority-Muslim country. However, given that these mechanisms of social change have yet to be compared across different societies on many variables, an initial comparison – albeit limited – is valuable.

We also selected variables for our analysis based on relevance and completeness. In terms of the former, we choose variables that reflect cultural attitudes that could plausibly change over time. This includes a wide range of items, from opinions about child-rearing to attitudes about the acceptability of euthanasia. We also select variables based on whether they have been asked in all the waves for the countries selected. After implementing both criteria we are left with 56 variables that cover a wide variety of issues, some mundane and some highly sensitive. The full list of items, alongside their respective questions and the abbreviations we use below, is available in the supplementary materials.

Given that we are interested in how sensitive (or not) these questions are, we fielded a multi-country survey to measure sensitivity. Although operationalising sensitivity is difficult, we find the definition given in this special issue a useful starting point. As mentioned above, we follow Campbell and Mace (this issue) in defining sensitive topics as topics that are difficult to talk about. We took this definition and asked respondents to tell us how easy or difficult it would be to discuss our 56 survey questions from the WVS.

Importantly, we are not interested in the respondents’ own opinions on a given issue, but rather on how difficult they think it would be for the *majority of their compatriots* to talk about that question. Thus, we asked them: ‘how difficult would it be for the majority of people from you country to discuss the following question’. We then provided them with a scale from 1 to 10, where 1 was labelled ‘not difficult at all’ and 10 was labelled ‘extremely difficult’. Each participant rated all 56 questions. This provides a plausible measure of how sensitive each issue is in each of the eight countries in our survey sample.

To field the surveys, we used the online platforms Prolific and CloudResearch. Both offer a high-quality pool of respondents across different countries (Peer et al., 2017). Using both platforms, we were able to reach respondents from the eight countries that comprise our WVS sample. We translated all the questions to the main languages spoken in each country, and we gave participants the opportunity to choose their preferred language. Initially, our sample consisted of 808 individuals and, after excluding participants that had missed more than two attention checks, we had total sample of 802 respondents. Table 1 breaks down how this sample is distributed across the countries.

**Table 1.** Sample size by country for the survey examining difficulty to discuss the WVS items.

Country	Number of Respondents
ARG	102
AUS	100
CAN	101
JPN	98
MEX	101
SWE	100
USA	100
ZAF	100

We do not claim that this sample is representative of the population of any of those countries. However, we believe that these data provide a principled measure of how sensitive certain issues are perceived in each country. The fact that we prompted participants to think about their second-order beliefs – to think about what most of their compatriots think – helps in trying to bypass individual idiosyncrasies and to get a adequate measure of country-level perceptions of sensitivity. This is reflected in the fact that the overall patterns in our data are plausible. Table 2, for example, shows the median score for the three issues that respondents, in each country, rated as the most difficult to discuss (we provide full descriptive results of the survey data in the Supplementary Materials).

**Table 2.** The three issues rated as the most sensitive in each country

Country	Median Difficulty	Variable
ARG		
	7.0	Confidence in government
	7.0	Justifiability of bribes
	7.0	Justifiability of euthanasia
AUS		
	7.0	Justifiability of suicide
	6.5	Justifiability of euthanasia
	6.0	Justifiability of abortion
CAN		
	7.0	Justifiability of euthanasia
	7.0	Justifiability of suicide
	7.0	Having a gay neighbor
JPN		
	7.0	Jobs for men over women
	7.0	Justifiability of abortion
	7.0	Justifiability of euthanasia
MEX		
	9.0	Justifiability of abortion
	8.0	Justifiability of euthanasia
	8.0	Justifiability of homosexuality
SWE		
	7.0	Jobs for nationals over foreigners
	7.0	Justifiability of suicide
	7.0	Having a neighbor of a different race
USA		
	8.0	Justifiability of suicide
	7.0	Justifiability of abortion
	7.0	Justifiability of euthanasia
ZAF		
	8.0	Justifiability of suicide
	7.0	Justifiability of abortion
	7.0	Justifiability of euthanasia

Unsurprisingly, the questions about the justifiability of abortion, euthanasia and suicide feature prominently in most countries, showing cross-national similarities in perceptions of sensitivity. However, we also pick up on some context-specific patterns: while issues of corruption and government credibility are perceived as difficult to discuss in Argentina, questions about race and immigration are seen as particularly sensitive in Sweden. This resonates with both the recent past of these countries and their current circumstances. The full descriptive summary of the data is provided in the supplementary materials, but overall the results seem to capture intuitive general trends while allowing for context-specific variation. While capturing the notion of sensitivity is challenging, we believe that our approach measures this concept reasonably well.

### Analysis

We begin our analyses by examining the relationship between *τ* and the total amount of change that each variable exhibits. For each variable, we calculate *τ*, as defined above, and then plot it against how much it has changed across the decades of observation (in standard deviations calculated by pooling the first and last waves). We then fit two regression models to explore whether the perceived sensitivity of an issue predicts how much it changes and the proportion of linear change attributable to between-cohort differences (*τ*). We use the median because it is less sensitive to extreme values, but our results show similar patterns when we use the mean sensitivity for each variable. In turn, the dependent variables of interest are, respectively, the absolute change exhibited by each variable in each country and the calculated *τ*. In both models, we allow for varying intercepts and slopes across countries to account for cultural variation – and similarities – between the different contexts.

## Results

**Figure 4.**
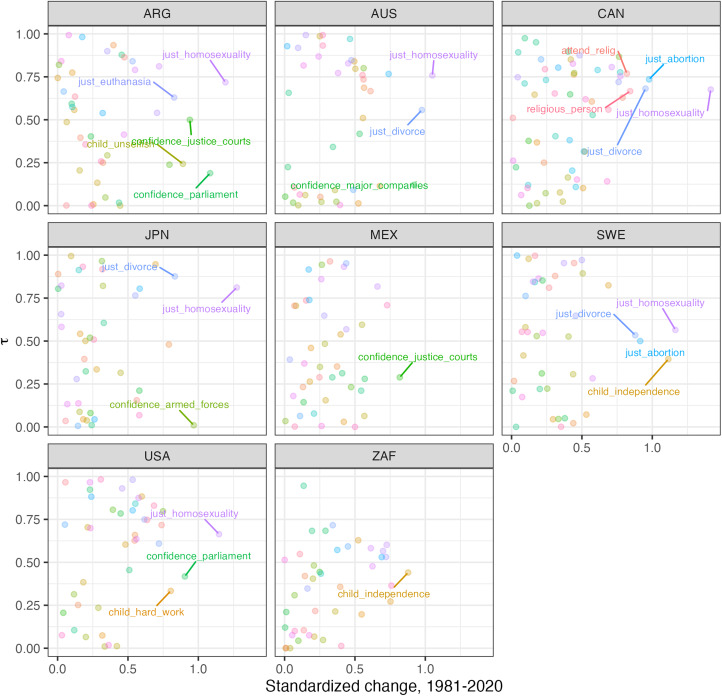
Relationship between the variance explained preserved when linear within-cohort changes are assumed to be zero and absolute change – in standard deviations – between the first and last wave.

We begin by calculating *τ* for all variables across each country. Figure 4 displays the relationship between *τ* on the *y*-axis and absolute change of the variable across the recorded time span on the *x*-axis.

On the *y*-axis, we have *τ*, which can be interpreted variance explained that is preserved when linear *within-cohort changes* are assumed to be zero. On the *x*-axis, we have the absolute change – in standard deviations – between the first and last wave. Therefore, in the upper right quadrant of each plot, we should see variables that have changed a lot and whose mean change can be well predicted by simple cohort replacement. In the lower right quadrant, we should see variables that have also exhibited a lot of change but whose variations are mostly accounted for by *within-cohort change*.

The first striking result is that most variables do not change much; most variables hover around the left-hand side of the x-axis. This is most evident in countries like Mexico and South Africa. In the plot, we label the variables that have displayed a directional change higher than 0.8 standard deviations.

Our results also seem to capture certain historical changes that we would expect given the time when the surveys were administered. For instance, changes in confidence in justice courts in Argentina coincide with the famous trials of the military dictatorship and the variation in confidence in the armed forces in Japan runs parallel with a restructuring of that institution.

Another important pattern that emerges is that, consistent with previous work on cultural change (Kiley & Vaisey, 2020; Tormos, 2021), the variable that seems to display consistently large change across countries is the justifiability of homosexuality. Notice that this variable tends to be on upper-right quadrant, which suggests that this change in mean over time is the result of *between-cohort differences*, with *within-cohort change* contributing a relatively small proportion of the change. In fact, at first glance, we see that this seems to be a common pattern for sensitive issues such as the justifiability of abortion, euthanasia and divorce. When they do exhibit considerable change, most of the variation is explained by cohort differences. The variables that exhibit change through *within-cohort change* are mostly related to confidence in institutions and childrearing. Thus, there seems to be a pattern in how change in different variables reflects different change mechanisms and it seems to be related to issue sensitivity.

We test the possible relevance of sensitivity directly in two ways. First, we examine whether the sensitivity of an issue is predictive of how much change it has undergone. Second, we analyse whether sensitivity predicts a variable's *τ*.

**Figure 5.**
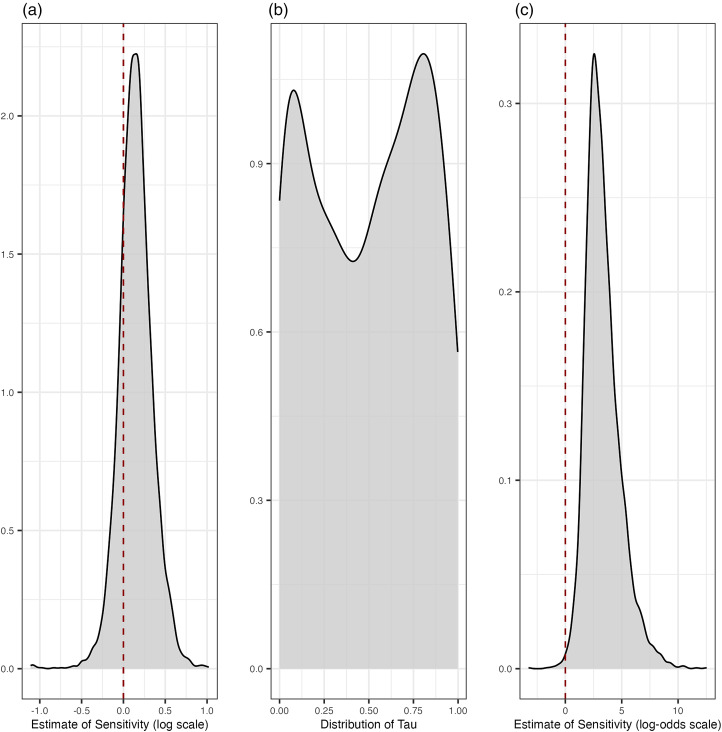
(a) Posterior estimate of the coefficient for sensitivity for the model regressing absolute change on sensitivity; (b) bimodality of tau; and (c) posterior estimate of the coefficient for sensitivity for the model regressing the probability of a value being drawn from the higher beta distribution on sensitivity.

To see if sensitive issues display different rates of overall change, we fit a linear regression model where the outcome is overall change and the main predictor is an issue's median sensitivity in each country. Given that the outcome variable is truncated at zero – a variable cannot display less than no change – we use the lognormal link. As mentioned above, the model includes varying intercepts and slopes at the level of country. Figure 5a shows the posterior distribution of the population-level coefficient for sensitivity in this model, on the log scale (a formal definition of the model, detailed results and assessments of fit are included in the Supplementary Materials). The distribution is centred around 0.15, and a considerable amount of the mass lies below 0. The uncertainty in this posterior distribution suggests that – in our data – there is not a strong relationship between an issue's perceived sensitivity and the amount of change.

**Figure 6.**
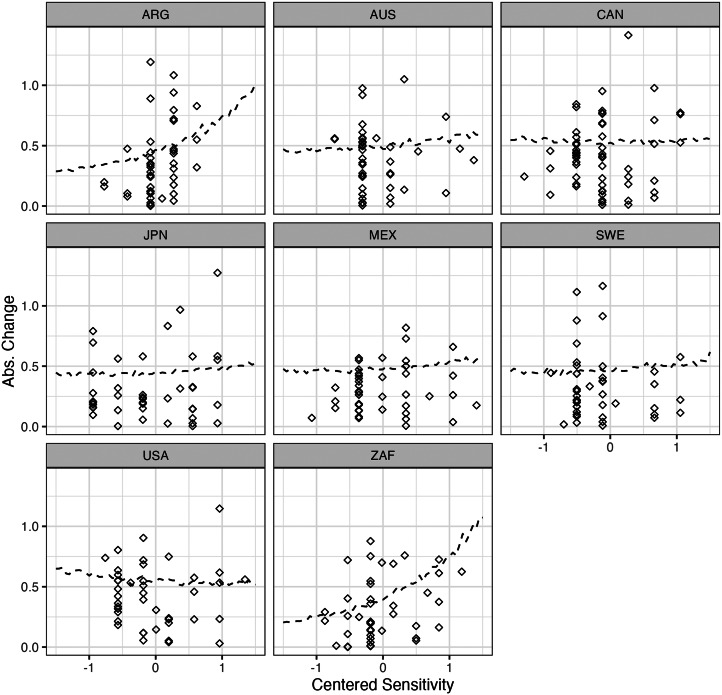
Model-implied average predictions for the model regressing absolute change in sensitivity.

In models with varying slopes and intercepts, it is difficult to interpret population-level effects, so it is more intuitive to plot model-implied predictions. Figure 6 shows these predictions with the *x*-axis representing centred sensitivity scales ranging from −1.5 standard deviations below the mean to 1.5 standard deviations above. The lines represent the mean prediction for each value of sensitivity. We notice that, while the relationship appears to be positive in countries like Argentina and South Africa, it is flat in the rest of our sample. Thus, we find no compelling evidence to suggest a specific relationship between an issue's sensitivity and the amount of aggregate change and, therefore, it is not possible to draw any strong conclusions.

To model *τ* as a function of sensitivity, it is necessary to make a few additional adjustments. First, given that *τ* is a proportion, it is bounded between 0 and 1. Second, in our data, *τ* exhibits a clear bimodality. We address this by fitting a finite-mixture model where we consider *τ* as produced by two different beta distributions, themselves bounded between 0 and 1. Figure 5b shows the bimodal distribution of *τ*, which we are going to model as two beta distributions.

Within the model, we also regress the probability of an issue belonging to the distribution with higher *τ* on that issue's perceived sensitivity. In other words, we ask the question: does the perceived sensitivity of an issue tell us whether it is more likely to have emerged from the beta distribution with higher average *τ*? If this is the case, then a higher sensitivity should be associated with a larger proportion of change explained solely by *between-cohort differences*. As above, in the regression component, we include varying intercepts and slopes at the level of country. It is worth noting that we also perform this analysis using Gaussian linear regression and the results are substantially the same. We include those analyses in the Supplementary Materials.

Figure 5c displays the posterior distribution for the population-level coefficient for the effect of sensitivity on the probability of belonging to the distribution with higher *τ* (a formal definition of the model, detailed results and assessments of fit are included in the Supplementary Materials). The coefficient is in the log-odds scale, but it is readily apparent that the majority of its mass lies above 0. The model suggests then that as an issue's sensitivity increases, so does the probability that it belongs to the distribution with higher average *τ*.

**Figure 7.**
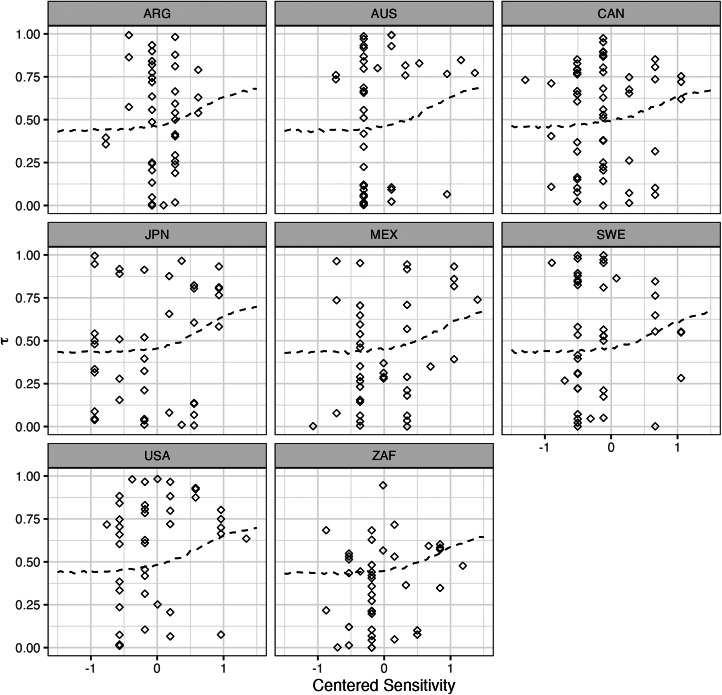
Model-implied average predictions for the model regressing tau on sensitivity.

However, results in the log-odds scales are notoriously difficult to interpret. Given the overall complexity of the model, it is better to examine its predictions to understand the implications of the results. Figure 7 displays the model's predicted outcomes, where the lines represent the average prediction at each value of sensitivity. We notice that the slopes consistently exhibit a slight, positive relationship. There is a small amount of variation between countries; for example, while a one standard deviation increase in sensitivity predicts an increase in *tau* of 0.158 in Mexico, it predicts and increase of 0.147 in Sweden. Despite this variation, the evidence for a positive relationship is consistent across countries. Thus, although the effect of sensitivity is not large, our model suggests that – on average – we should expect more sensitive issues to change more through *between-cohort differences* rather than via *within-cohort changes*.

## Discussion and conclusion

In this paper, we expanded on previous research on cultural change by investigating mechanisms that underlie processes of large-scale cultural change. Our investigation led to several relevant findings.

First, we found that most beliefs do not exhibit a large degree of linear change, even over four decades. As previous research has shown, across most countries, attitudes towards homosexuality do show considerable change (Tormos, 2021). A few other variables show large changes as well but they vary from country to country without a clear pattern.

Second, we found that a considerable proportion of linear change on many issues can be approximated well by a simple model that assumes that cohort succession is the only mechanism that can produce linear belief changes over time. The justifiability of homosexuality is one of these variables, indicating that the linear change in beliefs on this issue can mostly be mostly attributed to between-cohort differences. In some countries, we see a similar pattern for other sensitive issues like attitudes around divorce and euthanasia (Tormos et al., 2023).

Third, our models captured important historical contingencies that produced major within-cohort changes, such as the restructuring of the armed forces in Japan or the trials of the military dictatorship in Argentina. This provides some additional confidence that our models are not stacking the deck in favour of cohort replacement mechanisms.

Lastly – and most important – we found that, although beliefs about sensitive issues do not change more than beliefs about mundane issues, they do seem to change more via cohort replacement. This provides an interesting window into one issue-specific mechanism that might influence how cultural change happens. We cannot know whether this pattern is the result of beliefs on sensitive issues being deeply held, because people cannot gain accurate information about the beliefs of other people, or for some other reason, but this pattern is worthy of future study.

It is worth considering how our findings relate to other recent work on cultural change. At first glance, our results may seem at odds with the work of Tormos (2021), who emphasises within-cohort change. However, we think that there is more common ground than it appears. We replicate his finding that attitudes towards homosexuality have changed considerably across most contexts. While the *τ* for this variable tends to be high across countries, it is not at its theoretical maximum. This means that some within-cohort change is happening everywhere, which he illustrates clearly in his work.

Consider Tormos's (2021) example of within-cohort changes in beliefs about homosexuality in Sweden, which he argues have been substantial. Our analyses echo his findings in that we find that the relative contribution of between-cohort differences and within-cohort change is fairly equal. In the US, *τ* is around 0.67, meaning that within-cohort changes play a considerable role in accounting for linear change. Thus, our work replicates some of his main findings: that beliefs about the justifiability of homosexuality display considerable linear change and that within-cohort changes are a key part of that variation. We approach the question of relative importance from a slightly different angle, however; while Tormos focuses on coefficient sizes, we examine the relative explanatory contribution of allowing within-cohort change vs. restricting it to zero. We believe that our method compels us to think about the relative explanatory power of each mechanism. Even under the circumstances of steep within-cohort changes, between-cohort differences could remain the primary mechanism of long-term linear change.

Overall, we believe that this study contributes to ongoing conversations about the mechanisms of cultural change. We contend that, at the heart of current debates about cultural change lies the question of the relative explanatory power of within-cohort changes and between-cohort differences. Although we found strong evidence that between-cohort differences and cohort succession are important mechanisms of directional cultural change, we cannot make progress by simply counting the number of variables that seem to be better explained by one process or another. Nor do we simply want to say ‘it depends’ when we consider the relative importance of change mechanisms. Future work will need to look at characteristics of issues themselves to better understand which change mechanisms are more likely to apply. We hope that our investigation of sensitivity as one promising mechanism is a contribution to this endeavour.

## Supporting information

Restrepo Ochoa and Vaisey supplementary material Restrepo Ochoa and Vaisey supplementary material

## Data Availability

All the data we used in this paper is available in the following link: https://github.com/NicolasRestrep/sensitive_change. We also include all the code necessary to reproduce all the analyses in this paper.

## Supplementary material

To view supplementary material for this article, please visit https://doi.org/10.1017/ehs.2024.13

## Research transparency and reproducibility

We are committed to making our paper as transparent and reproducible as possible. All data and code necessary to reproduce this paper – as well as the supplementary materials – can be found here: https://github.com/NicolasRestrep/sensitive_change
