# Supplementary material for: Opinions on hard-to-discuss topics change more via cohort replacement
Source: Evol Hum Sci. 2024 Apr 8;6:e25. doi: 10.1017/ehs.2024.13 (PMC11058531; doi:10.1017/ehs.2024.13)

Opinions on Hard-to-Discuss Topics Change More via Cohort Replacement

Nicolas Restrepo Ochoa, Anthropology Department, Univerity of California at Davis.

Stephen Vaisey, Sociology Department, Duke University.

## Questions & Abbreviations

1. Please tell me whether family is very important in your life, not important at all in your life, or something in between. (important_family)
2. Please tell me whether friends are very important in your life, not important at all in your life, or something in between. (important_friends)
3. Please tell me whether leisure is very important in your life, not important at all in your life, or something in between. (important_leisure)
4. Please tell me whether politics are very important in your life, not important at all in your life, or something in between. (important_politics)
5. Please tell me whether work is very important in your life, not important at all in your life, or something in between. (important_work)
6. Please tell me whether religion is very important in your life, not important at all in your life, or something in between. (important_religion)
7. There are many qualities that children can be encouraged to learn at home. Which, if any, do you consider to be especially important? (child_)
8. Would you rather not have people of a different race as neighbors? (neigh_diff_race)
9. Would you rather not have heavy drinkers as neighbors? (neig_drink)
10. Would you rather not have immigrants or foreign workers as neighbors? (neigh_imm)
11. Would you rather not have people with AIDS as neighbors? (neigh_aids)
12. Would you rather not have drug addicts as neighbors? (neigh_drugs)
13. Would you rather not have gay people as neighbors? (neigh_gay)
14. Would you say most people can be trusted or that you can never be too careful? (trust_people)
15. All things considered, how satisfied are you with your life as a whole these days? (life_satisf)
16. Some people feel they have completely free choice and control over their lives, while other people feel that what they do has no real effect on what happens to them. Please tell me whether you feel you have no choice and control at all over your life, a great deal of choice and control, or something in between. (choice_control)
17. Do you agree or disagree with the idea that when jobs are scarce, men should have more right to a job than women? (jobs_men_over_women)
18. Do you agree or disagree with the idea that when jobs are scarce, employers should give priority to people of this country over immigrants? (jobs_national_over_foreign)
19. Do you agree or disagree with the idea that being a housewife is just as fulfilling as working for pay? (housewife_fulfilling)
20. In political matters, people talk of “the left” and “the right.” How would you place your views on this scale, generally speaking? (politics_scale)
21. Would you say that incomes should be made more equal, that there should be greater incentives for individual effort, or something in between? (income_eq)
22. Would you say that businesses should be privately owned, publicly owned, or something in between? (pvt_state_owned)
23. Would you that the government should take more responsibility to ensure that everyone is provided for, that people should take more responsibility to provide for themselves, or something in between? (gvt_responsibility)
24. Would you say that competition is good, it is harmful, or something in between? (competition_good_evil)
25. How much confidence do you have in churches? (confidence_churches)
26. How much confidence do you have in the armed forces? (confidence_armed_forces)
27. How much confidence do you have in the press? (confidence_press)
28. How much confidence do you have in unions? (confidence_unions)
29. How much confidence do you have in the police? (confidence_police)
30. How much confidence do you have in parliament? (confidence_parliament)
31. How much confidence do you have in civil services? (confidence_civil)
32. How much confidence do you have in television? (confidence_television)
33. How much confidence do you have in the government? (confidence_government)
34. How much confidence do you have in political parties? (confidence_political_party)
35. How much confidence do you have in major companies? (confidence_major_companies)
36. How much confidence do you have in justice courts? (confidence_justice_courts)
37. How often do you attend religious services? (attend_relig)
38. Would you say you are religious, not religious, or an atheist? (religious_person)
39. Do you believe in God? (believe_god)
40. Do you believe in Hell? (believe_hell)
41. Please tell me whether God is very important in your life, not important at all in your life, or something in between. (important_god)
42. Please tell me whether you think claiming government benefits that you are not entitled to can always be justified, never be justified, or something in between. (just_gvt_benefits)
43. Please tell me whether you think avoiding a fare in public transportation can always be justified, never be justified, or something in between. (just_fare_public_trans)
44. Please tell me whether you think cheating on your taxes if you have a chance can always be justified, never be justified, or something in between. (just_cheat_taxes)
45. Please tell me whether you think accepting a bribe on the course of your duties can always be justified, never be justified, or something in between. (just_bribe)
46. Please tell me whether you think homosexuality can always be justified, never be justified, or something in between. (just_homosexuality)
47. Please tell me whether you think prostitution can always be justified, never be justified, or something in between. (just_prostitution)
48. Please tell me whether you think abortion can always be justified, never be justified, or something in between. (just_abortion)
49. Please tell me whether you think divorce can always be justified, never be justified, or something in between. (just_divorce)
50. Please tell me whether you think euthanasia always be justified, never be justified, or something in between. (just_euthanasia)
51. Please tell me whether you think suicide always be justified, never be justified, or something in between. (just_suicide)
52. How proud are you to be of the nationality of this country? (proud_nationality)

## Descriptive Summaries - Survey Data

The following plots present the distributions for the sensitivity scores in our data.

We will begin with the USA:


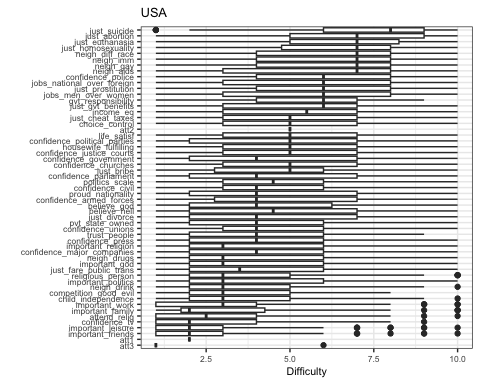


Let’s now look at Sweden:


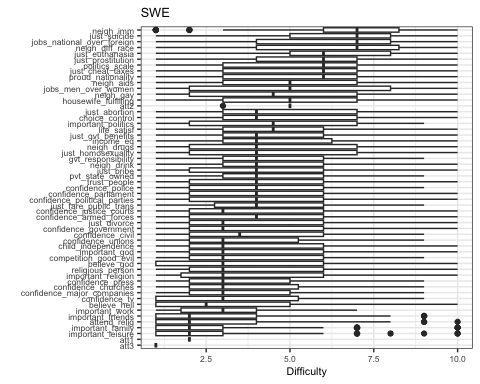


Let’s examine South Africa:


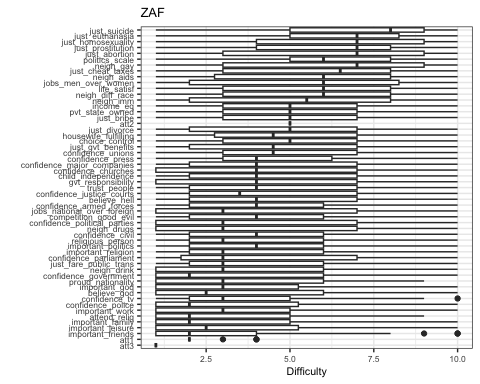


Let’s examine the responses for Mexico:


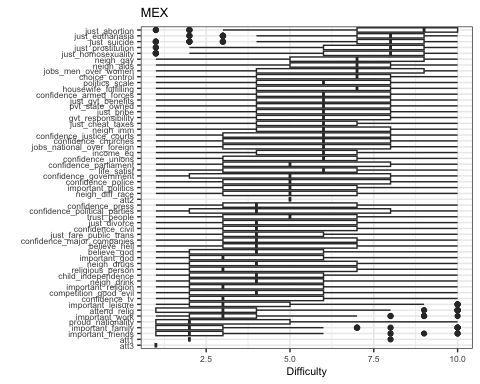


Let’s examine the responses for Japan:


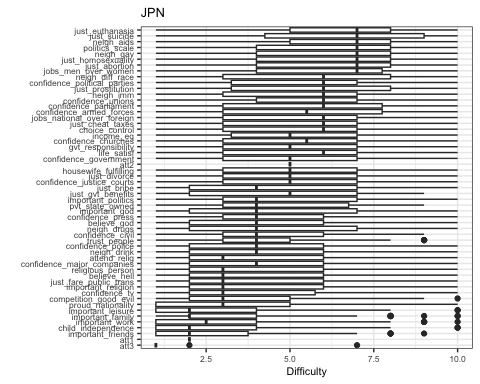


Now, let’s see the results from Canada:


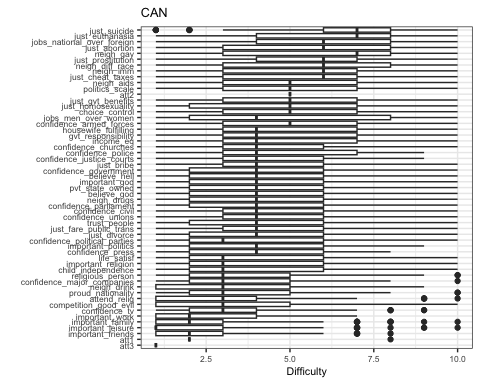


Let’s now look at Australia:


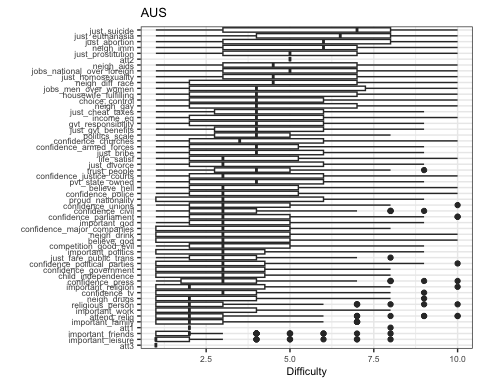


We finish by looking at the data from Argentina:


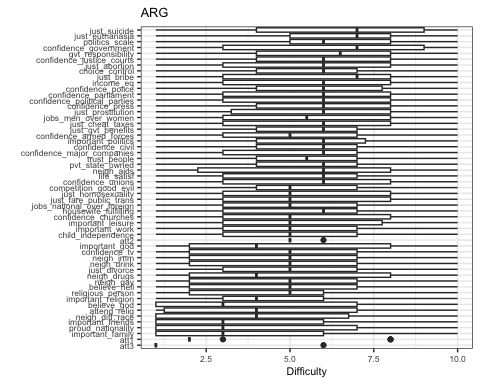


## Models

### Absolute Change and Sensitivity

We use this space to outline the specifications of the models we fit, as well as present the results in a more detailed manner. The first model we fit regresses the absolute change displayed by a variable on its sensitivity score. Given that the outcome variable has strictly positive values, we use the lognormal link. The model could be expressed as follows:

$$\text{Change}_{i}\sim\text{log-normal}\left( \mu_{i},\sigma\right)$$

$$\mu_{i}\sim\alpha_{\text{country}\left[ i \right]}+\beta_{\text{country}\left[ i \right]}S_{i}$$

$$\left( \begin{matrix} \alpha_{\text{country}} \\ \beta_{\text{country}} \end{matrix} \right) \sim\text{MVN}\left[ \left( \begin{matrix} \alpha_{\text{country}} \\ \beta_{\text{country}} \end{matrix} \right) ,S \right]$$

$$S=\left( \begin{matrix} \sigma_{\alpha} & 0 \\ 0 & \sigma_{\beta} \end{matrix} \right) R\left( \begin{matrix} \sigma_{\alpha} & 0 \\ 0 & \sigma_{\beta} \end{matrix} \right)$$

Table 1 displays the posterior estimates for the model:

| effect | component | group | term | estimate | std.error | conf.low | conf.high |
| --- | --- | --- | --- | --- | --- | --- | --- |
| fixed | cond |  | (Intercept) | -1.45 | 0.10 | -1.64 | -1.26 |
| fixed | cond |  | sensitivity | 0.15 | 0.20 | -0.24 | 0.56 |
| ran_pars | cond | country | sd__(Intercept) | 0.17 | 0.12 | 0.01 | 0.43 |
| ran_pars | cond | country | sd__sensitivity | 0.38 | 0.25 | 0.03 | 1.00 |
| ran_pars | cond | country | cor__(Intercept).sensitivity | -0.32 | 0.51 | -0.98 | 0.83 |
| ran_pars | cond | Residual | sd__Observation | 1.18 | 0.05 | 1.09 | 1.27 |

The posterior predictions for the model look reasonable:


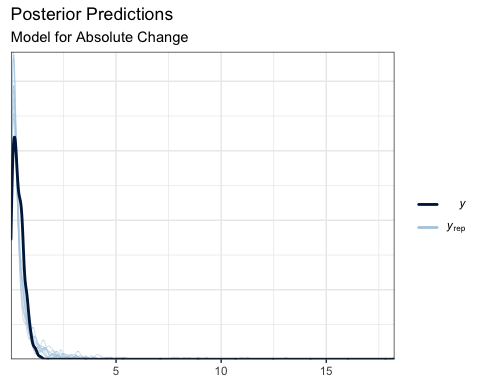


### Tau and Sensitivity

Before we explore the model that features on the main text, we will present the results for a model that uses gaussian linear regression to examine the relationship between $\tau$ and an issue’s sensitivity. Even though the conclusions are similar, the gaussian model exhibits poor ability to retrodict the data. Once we have established this shortcoming, the rationale behind the mixed model becomes clearer.

The gaussian linear model can be expressed as follows:

$$\tau_{i}\sim\text{Normal}\left( \mu_{i},\sigma\right)$$

$$\mu_{i}\sim\alpha_{\text{country}\left[ i \right]}+\beta_{\text{country}\left[ i \right]}S_{i}$$

$$\left( \begin{matrix} \alpha_{\text{country}} \\ \beta_{\text{country}} \end{matrix} \right) \sim\text{MVN}\left[ \left( \begin{matrix} \alpha_{\text{country}} \\ \beta_{\text{country}} \end{matrix} \right) ,S \right]$$

$$S=\left( \begin{matrix} \sigma_{\alpha} & 0 \\ 0 & \sigma_{\beta} \end{matrix} \right) R\left( \begin{matrix} \sigma_{\alpha} & 0 \\ 0 & \sigma_{\beta} \end{matrix} \right)$$

Table 2 displays the posterior estimates for the model:

| effect | component | group | term | estimate | std.error | conf.low | conf.high |
| --- | --- | --- | --- | --- | --- | --- | --- |
| fixed | cond |  | (Intercept) | 0.49 | 0.03 | 0.43 | 0.56 |
| fixed | cond |  | sensitivity | 0.08 | 0.04 | 0.00 | 0.16 |
| ran_pars | cond | country | sd__(Intercept) | 0.06 | 0.04 | 0.01 | 0.15 |
| ran_pars | cond | country | sd__sensitivity | 0.05 | 0.04 | 0.00 | 0.15 |
| ran_pars | cond | country | cor__(Intercept).sensitivity | -0.07 | 0.57 | -0.95 | 0.93 |
| ran_pars | cond | Residual | sd__Observation | 0.32 | 0.01 | 0.29 | 0.34 |

However, when we check the posterior predictions of the model, we notice considerable discrepancies with the data:


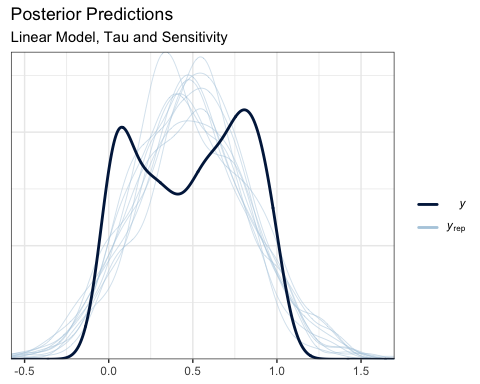


The data is clearly bimodal and our model is not capturing this. Our model mostly predicts values of $\tau$ in a region where they are quite sparse in our data.

As we explain in the text, a way to deal with this problem is to fit a mixture model. We are going to stipulate that the bimodal distribution pictured above is a combination of two distributions. In this case, we choose two beta distributions, because they are bounded between 0 and 1, just like our data.

Then, in the same model, we ask whether the sensitivity of an issue is associated with the probability of its $\tau$ being drawn from the beta distribution with higher values on average. If this is the case, then there is a positive relationship between an issue’s sensitivity and its $\tau$.

We could express the model as follows. We have two distirbutions, and we want to estimate the probability that a given value of $\tau$ is drawn from one or the other. Given that the outcome is binary, we can express it as a bernoulli trial (Franke, 2023):

$$g_{i}\sim\text{Bernoulli}\left( p \right)$$

Our two distributions – call them $0$ and $1$ – are two Beta distributions with their respective parameters:

$$g_{0}\sim Beta\left( \alpha_{0},\beta_{0} \right)$$

$$g_{1}\sim Beta\left( \alpha_{1},\beta_{1} \right)$$

Our central target our knowledge is: given a value of $\tau$, what distribution is it more likely to have come from?

$$\tau_{i}\sim Beta\left( \alpha_{g_{i}},\beta_{g_{i}} \right)$$

But then, we also examine whether the probability $p$ of being in a given group is associated with an issue’s sensitivity. As above, we include varying intercepts and slopes at the level of country:

$$g_{i}\sim\text{Bernoulli}\left( p \right)$$

$$\text{logit}\left( p_{i} \right)\sim\alpha_{\text{country}\left[ i \right]}+\beta_{\text{country}\left[ i \right]}S_{i}$$

$$\left( \begin{matrix} \alpha_{\text{country}} \\ \beta_{\text{country}} \end{matrix} \right) \sim\text{MVN}\left[ \left( \begin{matrix} \alpha_{\text{country}} \\ \beta_{\text{country}} \end{matrix} \right) ,S \right]$$

$$S=\left( \begin{matrix} \sigma_{\alpha} & 0 \\ 0 & \sigma_{\beta} \end{matrix} \right) R\left( \begin{matrix} \sigma_{\alpha} & 0 \\ 0 & \sigma_{\beta} \end{matrix} \right)$$

Table 3 displays the posterior estimates from the model:

| effect | component | group | term | estimate | std.error | conf.low | conf.high |
| --- | --- | --- | --- | --- | --- | --- | --- |
| fixed | cond |  | mu1_(Intercept) | -0.28 | 0.09 | -0.45 | -0.11 |
| fixed | cond |  | mu2_(Intercept) | 1.01 | 0.18 | 0.65 | 1.35 |
| fixed | cond |  | p_(Intercept) | -2.51 | 1.05 | -5.05 | -0.84 |
| fixed | cond |  | p_sensitivity | 3.32 | 1.52 | 0.95 | 6.97 |
| ran_pars | cond | country | sd__p_(Intercept) | 1.12 | 0.81 | 0.06 | 3.17 |
| ran_pars | cond | country | sd__p_sensitivity | 1.17 | 0.98 | 0.04 | 3.71 |
| ran_pars | cond | country | cor__p_(Intercept).theta2_sensitivity | -0.32 | 0.56 | -0.99 | 0.89 |

Though the substantial conclusions are not much different, the posterior predictive checks look more in line with the data:


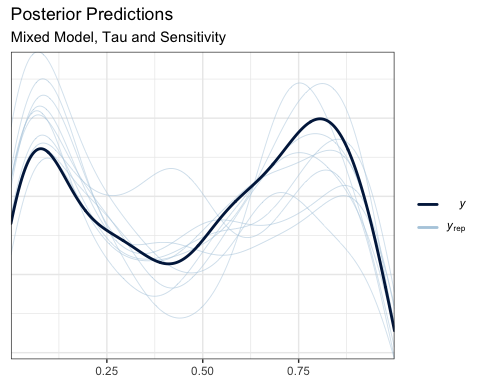

Supplement: Restrepo Ochoa and Vaisey supplementary material [file S2513843X24000136sup001.docx]
